# Antimicrobial Efficiency of Essential Oils from Traditional Medicinal Plants of Asir Region, Saudi Arabia, over Drug Resistant Isolates

**DOI:** 10.1155/2019/8928306

**Published:** 2019-01-17

**Authors:** Ismail M. Helal, Ashraf El-Bessoumy, Erwa Al-Bataineh, Martin R. P. Joseph, Prasanna Rajagopalan, Harish C. Chandramoorthy, Sami Ben Hadj Ahmed

**Affiliations:** ^1^Department of Clinical Laboratory Sciences, College of Applied Medical Sciences, King Khalid University, Abha, Saudi Arabia; ^2^Atomic Energy Authority, Nuclear Research Center, Plant Researches Department, P.N. 13759, Egypt; ^3^Department of Biochemistry, Faculty of Science, Alexandria University, Alexandria, Egypt; ^4^Department of Microbiology and Clinical Parasitology, College of Medicine, King Khalid University, Abha, Saudi Arabia; ^5^Laboratory of Protein Engineering and Bioactive Molecules 99 UR 09-26, National Institute of Applied Sciences and Technology, BP 676-1080 Tunis, Tunisia

## Abstract

Antimicrobial resistance (AMR) is a recurring global problem, which constantly demands new antimicrobial compounds to challenge the resistance. It is well known that essential oils (EOs) have been known for biological activities including antimicrobial properties. In this study, EOs from seven aromatic plants of Asir region of southwestern Saudi Arabia were tested for their antimicrobial efficacy against four drug resistant pathogenic bacterial isolates (*Staphylococcus aureus, Streptococcus pyogenes, Escherichia coli, *and* Streptococcus typhimurium) *and one fungal isolate (*Candida albicans)*. Chemical compositions of EOs were determined by gas chromatography-mass spectrometry (GC-MS). The results revealed that EOs from* Mentha cervina*,* Ocimum basilicum,* and* Origanum vulgare *proved most active against all isolates with inhibitory zone range between 17 and 45 mm. The lowest minimum inhibitory concentration (MIC) of 0.025mg/ml was observed for* Staph. aureus* and* Streptococcus pyogenes* with EO of* Origanum vulgare*. All the three EOs showed significant anticandida activity. The results related to EOs from* Mentha cervina*,* Ocimum basilicum,* and* Origanum vulgare *demonstrated significant antimicrobial efficacy against drug resistant microorganisms.

## 1. Introduction

Drug resistance is a recurring phenomenon that demands utmost medical attention worldwide [1]. The use of antibiotics is a major strategy for the eradication of pathogen bacteria and antimicrobial agents are commonly used therapeutically and prophylactically in human medicine therapy [2]. However, increased resistance to these drugs is an inevitable side effect. Thus, the unsuitable use and overprescription of antibiotics in human medicine therapy are considered to be the main sources for the expansion and spread of bacterial resistance to antibiotics; even very low concentrations of antibiotics released into the environment can enrich the population of resistant strains [3, 4]. Otherwise, the wide use of antibiotics increases the probability of unfavorable infection outcome in the last years with the intensification in the prevalence of infections caused by drug resistant strains [5, 6].

One of the recommended methods to deal with drug resistant bacteria is the usage of natural alternative remedies for the cure of numerous infectious diseases, which include natural antimicrobial constituents such as natural plant compounds. Plant-based active compounds are among the alternative agents tested in order to supply traditional antibiotics and synthetic antimicrobials [7] and are considered a significant source of new chemical substances with potential therapeutic effects [8, 9]. Essential oils (EOs) from aromatic and/or medicinal plants, which constitute the odorous, volatile products of an aromatic plant's secondary metabolism, have been used for many years all over the world in either food processing as flavor enhancers, preservatives, remedies, and cosmetics [10, 11].

In medicine, EOs have been researched for their antibacterial, antifungal, antiviral, insecticidal, anticancer, and antioxidant properties [11, 12]. Particularly, the EOs that possess antimicrobial activities have been the subject of many scientific reports resulting in the screening of a wide variety of plant species [13–15]. The main advantage of these natural products is that they do not increase antibiotic resistance with the long-term medicinal usage. They tend to have low mammalian toxicity, less environmental effects, and wide public acceptance [16]. On the other hand, the chemical composition of the EOs that contribute to their medicinal value which is responsible for the antibacterial properties highly depends on various factors like the climatic and geographical conditions, as well as harvesting, isolation techniques, and storage [13].

Medicinal plants like* Mentha cervina*,* Ocimum basilicum*,* Mentha pulegium *L,* Origanum vulgare, and Salvia officinalis* belong to the* Lamiaceae* family. This family is one of the most important of its kind which is used in the production of essential oils with antioxidants and antimicrobial properties [17]. They are widely used in folk medicine for the treatment of many digestive tract diseases and in culinary arts [18].* Ruta graveolens*, a member of* Rutaceae* family, has been demonstrated widely for its therapeutic usage in the traditional medicine, which includes microbial infection, menstrual disorders, skin inflammations, cramps, earache, and headache [14, 19, 20]. Another plant of this category is* Scirpoides holoschoenus*, belonging to* Cyperaceae* family. It was noted that Scirpoides genus is a medicinally important group containing various secondary metabolites and its antimicrobial activity has not been reported till date [21].

The purpose of the present study is to examine the effect of EO extracts of* Mentha cervina*,* Ocimum basilicum*,* Ruta graveolens*,* Mentha pulegium L*,* Origanum vulgare*,* Scirpoides holoschoenus,* and* Salvia officinalis* on drug resistant Gram-positive and Gram-negative bacteria and further elucidate bacteriostatic/ bactericidal concentrations of these oils. The study also aims to compare the active concentrations of EOs with established anti-microbial agents against these drug resistant microbes to draw out a direct comparison in terms of efficacy.

## 2. Materials and Methods

### 2.1. Plant Materials

Ariel parts of* Mentha cervina, Ocimum basilicum, Ruta graveolens, Mentha pulegium L, Origanum vulgare, Scirpoides holoschoenus,* and* Salvia officinalis* were collected at the time of flowering stage from Asir region located in the southwestern part of Saudi Arabia. The collected plants were subjected to scientific identification using the scientific identification manuals with the kind help of our botany specialized colleagues in the Biology Department, Faculty of Science, King Khalid University. The samples were shade-dried for 10 days before the steam distillation.

### 2.2. Isolation of the Essential Oils

The air-dried, finely grounded raw material (Table 1) was submitted to hydrodistillation in a Clevenger-type apparatus. Obtained EOs were then dried over anhydrous sodium sulphate, filtered, and stored at 4°C until use.

### 2.3. Chemical Composition of Essential Oils

The GC-MS analysis of EOs was carried out on a Thermo Scientific TRACE 1310 Gas Chromatograph attached with ISQ LT single quadruple Mass Spectrometer, equipped with DB-5 column (30 m × 0.32 mm, i, d., 0.25 *μ*m film thickness, J&W Scientific). The ionization mode is EI with electron ionization energy of 70 eV. The temperature of the column was programmed from 40°C to 275°C at 5°C/min. The injector and detector temperatures were the same at 300°C. Helium was used as the carrier gas at a flow rate of 1.0 ml/min. The identification of the chemical compounds was based on mass spectra (Wiley 275.L, 8th edition mass spectral library), or with standards when available, and confirmed by comparison of their GC retention indices either with those of standards or with data published in the literature as described by Adams (2007) [22].

### 2.4. Culture Conditions for Drug Resistant Microbes

The bacteria and fungi were isolated from clinical specimens obtained from Asir hospital through proper channel for research. These isolated organisms were confirmed for antibiotic resistance by antibiotic sensitivity pattern and were determined by Kirby-Bauer method [23]. The EOs were individually tested against a panel of microorganisms. Two Gram-positive bacteria,* Staphylococcus aureus* and* Streptococcus pyogenes*, and two Gram-negative bacteria,* Escherichia coli* and* Salmonella typhimurium* were chosen for the study. The bacterial strains were cultivated in Luria-Bertani Medium (LB) (Oxoid Ltd, UK) at 37°C. Working cultures were prepared by inoculating a loopful of each test bacteria in 3 ml of Muller–Hinton broth (MH) (Oxoid Ltd, UK) and were incubated at 37°C for 12 h. For the test, final inoculum concentrations of 10^6^ CFU/ ml bacteria were used.

*Candida albicans* was cultured in Sabouraud dextrose broth (SDB) or on Sabouraud dextrose agar (SDA) (Difco, Sparks, MD, USA) for 48 h at 35°C. A standardized inoculum isolate of Candida was propagated in SDB at 35°C for 24 h with 200 rpm agitation. One ml of 24 h old culture in SDB was centrifuged (3900 rpm at 4°C for 1 min), and the pellets were washed twice with 1 ml of physiological saline. Sterile physiological saline was added to give a McFarland turbidity of 0.5 at 530 nm, corresponding to 5 × 10^6^CFU /ml).

### 2.5. Antibiotic and Antifungal Standards

The antibiotics and antifungals drugs (RA-rifampicin: 5 *μ*g, DA-clindamycin: 2 *μ*g, P-penicillin G: 10 IU, CLR-clarithromycin: 15 *μ*g, AMX-amoxicillin: 25 *μ*g, MTZ-metronidazole: 5 *μ*g, OXA-oxacillin: 5 *μ*g, TMP-trimethoprim: 5 *μ*g, SXT-trimethoprim-sulfamethoxazole: 1.25*μ*g/23.75*μ*g VAN-vancomycin: 30 *μ*g, AMB-amphotericin: B: 100 *μ*g, FLC-fluconazole: 25 *μ*g, and KTC-ketoconazole: 15 *μ*g) were used as positive control. The logic of selection of these antibiotics was based on the antibiotic susceptibility testing.

### 2.6. Disc-Diffusion Method

The paper disc-diffusion method was employed for the determination of antimicrobial activities [24]. Briefly, suspension in LB or SDB of the tested microorganism (0.1 ml of 10^7^–10^8^ cells per ml) was spread on the solid media plates. Paper discs (9 mm in diameter) were impregnated with 12 *μ*l of the oil and placed on the inoculated plates. These plates, after remaining at 4°C for 2 h, were incubated at 37°C for 24 h for bacteria and 48 h at 35°C for* Candida*. The diameter of the inhibition zones was measured in millimeters. All the tests were performed in triplicate and repeated twice. These data were compared to antibiotics and antifungals with recommended doses used as positive controls tested and in the presence of negative control.

### 2.7. Determination of the Minimum Inhibitory Concentration (MIC)

Microdilution method was used for determination of MIC of the EOs. All tests were performed in LB or SDB, complemented with DMSO (highest final concentration 0.1%). Microbial strains were cultured at 37°C overnight in LB or SDB. Test strains were suspended in LB or SDB to provide a final density of 5 × 10^5^ CFU/ml and these were confirmed by viable counts. Regular dilutions ranging from 0.0125 mg/ml to 200 mg/ml of the essential oil were prepared in 96-well microtiter plate (Iwaki brand, Asahi Techno Glass, Japan), including one growth control (LB+DMSO) and one sterility control (tested oil+ LB+DMSO). Plates were incubated under normal atmospheric conditions at 37°C for 24 h under vigorous agitation for bacteria and 48 h at 35°C for* Candida*. The wells were then examined for indication of growth and MIC values were determined as the lowest EO concentration that inhibited visible growth of the tested microorganism which was indicated by the presence of a white “pellet” on the well bottom. The negative controls were set up with DMSO in amounts corresponding to the highest quantity present in the test solution (0.1%). The tests were performed in triplicate and repeated twice. These data were compared to antibiotics with recommended doses used as positive controls tested and in the presence of negative control.

### 2.8. Determination of the Minimal Bactericidal and Fungicidal Concentrations (MBC and MFC)

The wells with no visible growth were selected and samples were used to determine the MBC and MFC. Briefly, after homogenization, a loop of each suspension was cultured on LB agar or SDA, respectively. This culture was incubated aerobically at 37°C for 24 h for bacteria or 48 h at 35°C for* Candida*. The MBC or MFC of each was estimated from the culture medium in which no visible microbial growth was recorded upon examination. The tests were performed in triplicate and repeated twice

### 2.9. Statistical Analysis

All experimental results of antibacterial and antifungal experiments were repeated three times and are expressed as mean ± standard deviation (SD).

## 3. Results 

### 3.1. Antimicrobial Activity of EOs by Disc-Diffusion Method

In the present study, the* in vitro* antimicrobial activity of different EOs against the studied microorganisms (*Staphylococcus aureus*,* Streptococcus pyogenes*,* Escherichia coli*,* Salmonella typhimurium*, and* Candida albicans*) and their activity potentials was quantitatively assessed by the presence or absence of inhibition zones using the agar disc-diffusion method. These data were compared to standard antibiotics and antifungals with recommended doses used as positive controls as mentioned above and in the presence of negative control. The results showed great variations in the potency of the antimicrobial activity of selected species.

In fact, it can be noted from Tables 2(a) and 2(b) that the EOs of* Mentha cervina*,* Ocimum basilicum,* and* Origanum vulgare* were found to have highest activities against all the tested microorganisms studied, and the size of their inhibition zones varied between 19 and 45 mm. The strong antibacterial activity of these EOs may be attributed to the presence of bioactive metabolites of various chemical types, such as Pulegone (58.54%), L-Linalool (60.97%), and 1-Terpineol (19.68%) of* Mentha cervina*,* Ocimum basilicum,* and* Origanum vulgare*, respectively. However, essential oil (EO) of* Mentha pulegium L* revealed a moderate activity against all tested strains (from 13 to 45 mm). Further, our results showed that EO of* Salvia officinalis* was not active against any tested strains except a small inhibition against* Salmonella typhimurium* and against* Streptococcus aureus* with inhibition zones of 10 and 13 mm, respectively. Also EOs of* Ruta graveolens* and* Scirpoides holoschoenus* showed total lack of antimicrobial activity against* Escherichia coli* and* Salmonella typhimurium* with a moderate activity against remaining strains. Interestingly, all antibiotics showed low inhibition zone (from 0 to 13 mm) with recommended doses which confirms the efficacy of these EOs over standard antibiotics.

### 3.2. MIC, MBC, and MFC

From the maxim inhibition zones observed with disc-diffusion method, the MIC values were determined by the microdilution broth assay for* Mentha cervina*,* Ocimum basilicum,* and* Origanum vulgare* (Table 3). The results of the MIC, MBC, and MFC values against tested Gram-positive, Gram-negative bacteria, and* Candida albicans* varied from 0.025 to 25 mg/mL and from 0.05 to 50 mg/mL, respectively. Our results suggested that* Origanum vulgare* EO possessed the highest inhibitory effect against* s. aureus* with MIC and MBC values of 0.025 and 0.05mg/ml, respectively, followed by EOs of* Mentha cervina* and* Ocimum basilicum*.

In* Staphylococcus pyogenes*, the best effect was also related to* Origanum vulgare* EO with MIC and MBC of 0.025 and 0.05mg/ml, respectively. Results from MIC and MBC of the EOs on* Escherichia coli* and* Salmonella typhimurium* revealed that the highest inhibitions were again from* Origanum vulgare*. Best active EO against* Candida albicans* was from* Mentha cervina* which had MIC and MFC of 0.4mg/ml and 0.8mg/ml, respectively. However, it was observed that the antibacterial activity of these EOs depends on its concentration and the tested bacteria strain. Interestingly, the three selected EOs exhibited a stronger antibacterial activity against Gram-positive (0.025mg/ml–1.6 mg/ml) than Gram negative (0.2–12.5 mg/ml) bacteria. Th*e Candida albicans* was identified as the most resistant strain with MIC and MBC equal to 0.4 and 25 mg/ml, respectively.

### 3.3. Chemical Composition of the Essential Oils

EOs of most active plants (*Mentha cervina, Ocimum basilicum,* and* Origanum vulgare*) were obtained by hydrodistillation and analyzed by GC-MS to determine their chemical composition. Results are presented in Tables 4, 5, and 6.

According to Table 4, a total of twenty-four active components were identified for* Mentha cervina*. Pulegone (58.54%) was identified as the main active compound. Following this, 1-Menthone (6.91%), Eucalyptol (6.76%), and L-Linalool (6.44%) were also detected in the* Mentha cervina* oil. In addition, Estragole, endoborneol, Bicyclo[3.1.1]heptane,6,6-dimethyl-2-methylene, Isopulegol, D-Limonene, and Alpha-Pinene were detected with the concentrations of 2.70, 2.31, 1.90, 1.46, 1.21, and 1.05%, respectively. The other components were found to be the minor components in the essential oil.

In* Ocimum basilicum* EO, twenty-three compounds were characterized and identified (Table 5). The major constituents were represented by L-Linalool (60.97%) and Estragole (21.56.3%). Other compounds were detected in relatively low concentrations such as Pulegone (4.21%), Eucalyptol (2.28%), Cyclohexanone,5-methyl-2-(1-methylethyl)-,cis-(CAS) (1.79%), Trans- alpha-Bergamotene (1.57%), Cyclohexanone,5-methyl-2-(1-methylethylidene), (1.52%), Alpha-Fenchyl acetate (1.17%), l-Menthone (1.16%), Camphor (0.81%), and Germacrene-D (0.66%). In addition, some components were detected in trace percentages.

Data of GC and GC-MS analyses for* Origanum vulgare *EO are shown in Table 6. It is obvious from the data that thirty-six compounds accounting for 99.99% of the extracted EO were identified. 1-Terpineol (19.68%), Sabinene (17.17%), Gamma-Terpinene (12.99%), Alpha-Humulene (CAS) (10.57%), Alpha-Phellandrene (9.18%), Cis-trans Sabinene hydrate (6.13%), Cis-sabinene hydrate acetate (6.13%), and 3-Cyclohexen-1-ol,4-methyl-1-(1-methylethyl)-(CAS) (5.64%) are the predominant components of the EOs. While Alpha-Myrcene, cis-Ocimene, Alpha-Terpinolene, and p-Cymene resulted in a relatively low ratio with respective percentages of 3.44, 2.74, 2.31, and 1.35%, respectively, the other components were detected in trace ratios.

## 4. Discussion

The research of alternative and effective drugs from medicinal plants against drug resistant antibiotics has become a priority concern all over the world. The overuse of antibiotics is considered a main cause for antibiotic resistance [16]. All EOs displayed highly varying MIC and MBC values against resistant microorganisms tested but the highest values belong to* Mentha cervina*,* Ocimum basilicum,* and* Origanum vulgare*. Our results are in concordance with previous studies revealing the potential of EOs to exhibit strong activity against drug resistant bacteria [2, 4, 5, 25]. This resistance is due to intrinsic factors and can be transferred to susceptible strains during horizontal genetic transfer, particularly in hospital environment. Our promising findings provide evidence that EO from medicinal plants of Asir region of Saudi Arabia exhibits efficacy against drug resistant pathogenic microorganisms and they will be clinically valuable.

To our knowledge, there are no data in the literature to evaluate antimicrobial activities of EOs extracted from medicinal plants distributed in Asir district of Saudi Arabia against drug resistant pathogenic microorganisms. All EOs revealed antibacterial properties, but the degree of bacterial growth inhibition induced by plant materials was shown to be related to bacterial strain and herbal source [13]. Observations from disc-diffusion method inferred that herbal EOs had variable inhibition zones both on Gram-positive (*S. aureus *and* S. pyogenes*) and on Gram-negative bacteria (*E. coli *and* S. typhimurium*). Interestingly, the most active EOs belong to the* Lamiaceae* family. EOs of* Mentha cervina*,* Ocimum basilicum,* and* Origanum vulgare* exposed almost identical antimicrobial potentials. These results are in accordance with the earlier findings [18, 26] that EOs extracted from* Lamiaceae* family showed the highest antimicrobial activity.

All species from* Lamiaceae* family exhibited the strongest antifungal activity with inhibition zones ranging from 39 to 45 mm compared with standard antifungal molecules (Ketoconazole, Fluconazole, and Amphotericin 100) which range from 0 to 14 mm only. Previous studies have already shown a highly effective inhibition by* Ocimum gratissimum* EO on pathogenic fungi* Aspergillus spp., Candida spp., Malassezia spp., Cryptococcus spp., Sporothrix spp., Microsporum spp., and Trichophyton spp.* [27, 28].

The results of MIC and MBC obtained in this study confirmed our earlier observations of Gram-positive bacteria being more susceptible to growth inhibition by plant EOs than Gram-negative bacteria [8, 9, 13]. These differences could be attributed in part to the great complexity of the double membrane-containing cell envelope in Gram-negative bacteria compared to the single membrane structure of the positive ones [9]. These differences may also be attributed to the presence of the lipopolysaccharides in the outer membrane of the Gram-negative bacteria, which provides a hydrophilic surface and functions as a permeability barrier for many plant extracts, antibiotics, detergents, and lipophilic compounds [9, 13]. However, the ability of EOs to disrupt the permeability barrier of cell membrane structures and the accompanying loss of chemiosmotic control is the most likely reason for its lethal action [25]. It is believed that the EOs can coagulate the cytoplasm and damage lipids and proteins [3].

For the chemical composition of* Mentha cervina*, these results are in accordance with previous studies of Rodrigues et al. [29] who found that the chemical composition of* Mentha cervina* EOs were dominated by the monoterpenes pulegone (52–75%), isomenthone (8–24%), limonene (4–6%), and menthone (1–2%).

We obtained the same tendency with* Ocimum basilicum*. In fact, our results are in good agreement with those of Hussain et al., [30] who reported that linalool was the main component in* O. basilicum* EO grown in Pakistan. Also, Gurbuz et al. [31] found that linalool (41.2%) was the main compound, identified in the hydrodistilled* O. basilicum* EO from Turkey. While, Purkayastha and Nath [32] reported that the major components in* O. basilicum* EO from northeast India were limonene, camphor, and b-selinene.

In contrast, the major constituents of* Origanum vulgare *were different either in composition or in concentration compared to previously published data [33, 34].

Taken together, the observed differences in the constituents of essential oils across countries may be due to different environmental and genetic factors, different chemotypes, and the nutritional status of the plants. Generally, antimicrobial activities of the EOs are difficult to correlate with a specific compound due to their complexity and variability; nevertheless, some investigators stated that there is association between the chemical composition of the most predominant components in the EO and the antimicrobial activity [13, 35].

## 5. Conclusions

In conclusion, we have identified new essential oils from the medicinal plants of Saudi Arabia, Asir region, which have demonstrated excellent anti-microbial activities against resistant pathogens. This study will add value to the medicinal properties of these herbs and urge these oils to be taken forward as novel agents against drug resistant microbes. However, more research is required to identify the active compounds of these plants to develop new antimicrobials.

## Figures and Tables

**Table 1:** List of plant species tested.

**No**	**Plant species**	**Family**	**Common Name**
**1**	*Mentha cervina*	*Lamiaceae*	Hart's pennyroyal
**2**	*Ocimum basilicum*	*Lamiaceae*	Basil
**3**	*Ruta graveolens*	*Rutaceae*	Rue
**4**	*Mentha pulegium*	*Lamiaceae*	Pennyroyal
**5**	*Origanum vulgare*	*Lamiaceae*	Oregano
**6**	*Scirpoides holoschoenus*	*Cyperaceae*	Scirpus
**7**	*Salvia officinalis*	*Lamiaceae*	Sage

All essential oils were obtained by hydrodistillation of the aerial parts of the plants.

**(a):** 

**Tested microorganisms**	**The tested essential oils**						
	***M. cervina***	***O. basilicum***	***R. graveolens***	***M. pulegium***	***O. vulgare***	***S. holoschoenus***	***S. officinalis***
***S. aureus***	30±1.83	20±2.58	13±0.82	14±1.41	24±1.15	21±1.63	13±0.82
***S. pyogenes***	19±1.83	19±1.63	9±0.82	13±1.83	18±1.15	8±1.41	0±0.0
***E. coli***	20±0.82	20±1.83	0±0.0	18±1.41	20±0.82	0±0.0	0±0.0
***S. typhimurium***	19±0.82	20±1.83	0±0.0	15±0.82	21±1.41	0±0.0	10±1.15
***C. albicans***	43±2.83	45±1.41	30±1.16	39±0.82	42±1.83	17±1.41	0±0.0

**(b):** 

**Tested microorganisms**	**P**	**RA**	**DA**	**CLR**	**AMX**	**MTZ**	**OXA**	**SXT**	**VAN**	**AMB**	**FLC**	**KTC**
***S. aureus***	0±0.0	10±0.82	9±0.82	12±1.15	8±0.82	0±0.0	13±1.42	10±0.82	15±1.16	ND	ND	ND
***S. pyogenes***	0±0.0	11±0.82	12±1.41	0±0.0	ND	0±0.0	ND	8±0.82	ND	ND	ND	ND
***E. coli***	0±0.0	13±1.41	0±0.0	0±0.0	0±0.0	0±0.0	0±0.0	0±0.0	0±0.0	ND	ND	ND
***S. typhimurium***	ND	0±0.0	0±0.0	7±0.82	11±0.82	0±0.0	0±0.0	9±0.82	0±0.0	ND	ND	ND
***C. albicans***	ND	ND	ND	ND	ND	ND	ND	ND	ND	14±0.82	0±0.0	0±0.0

ND: None Done

P: Penicillin G 10IU

RA: Rifampicin 5*μ*g

DA: Clindamycin 2*μ*g

CLR: Clarithromycin: 15*μ*g

AMX: Amoxicillin: 25*μ*g

MTZ: Metronidazole: 5*μ*g

OXA: Oxacillin: 5*μ*g

SXT: Trimethoprim-sulfamethoxazole: 1.25*μ*g/23.75*μ*g

VAN: Vancomycin: 30*μ*g

AMB: Amphotericin: B: 100*μ*g

FLC: Fluconazole: 25*μ*g

KTC: Ketoconazole: 15*μ*g

**Table 3:** The antimicrobial activities of tested essential oils (*Mentha cervina*, *Ocimum basilicum,* and* Origanum vulgare)* expressed by the Minimum Inhibitory Concentration (MIC in mg/ml) and minimal bactericidal and fungicidal concentrations (MBC in mg/ml and MFC in mg/ml) against tested microorganisms.

**Tested microorganisms**	***Mentha cervina***		***Ocimum basilicum***		***Origanum vulgare***	
	**(MIC)**	**MBC/MFC**	**(MIC)**	**MBC/MFC**	**(MIC)**	**MBC/MFC**
	**mg/ml**	**mg/ml**	**mg/ml**	**mg/ml**	**mg/ml**	**mg/ml**
***S. aureus***	0.05	0.1	1.6	3.2	0.025	0.05
***S. pyogenes***	1.6	3.2	0.05	0.1	0.025	0.05
***E. coli***	6.25	12.5	12.5	25	1.6	3.2
***S. typhimurium***	0.2	0.4	1.6	3.2	0.2	0.4
***C. albicans***	0.4	0.8	25	50	25	50

**Table 4:** Chemical composition of *Mentha cervina* essential oil determined by GC and GC- MS.

**No**	**Name of compounds**	**RT**	**Area **%
**1**	1-Butanol, 3-methyl-, formate	4.30	0.34
**2**	Propane, 2,2-diethoxy- (CAS)	4.65	0.32
**3**	Alpha -Pinene	10.87	1.05
**4**	Camphene	11.50	0.31
**5**	Sabinene	12.44	0.81
**6**	Bicyclo[3.1.1]heptane,6,6-dimethyl-2-methylene-,(1S)	12.56	1.90
**7**	Linalyl acetate	13.15	0.55
**8**	D-Limonene	14.52	1.21
**9**	Eucalyptol	14.62	6.76
**10**	6,9,12,15-Docosatetraenoic acid, methyl ester	14.84	0.10
**11**	Exo-2,7,7-trimethylbicyclo[2.2.1]heptan-2-ol	16.18	0.21
**12**	L-Linalool	17.24	6.44
**13**	Camphor	18.63	0.23
**14**	l-Menthone	18.92	6.99
**15**	p-Menthone	19.21	6.91
**16**	Endo-Borneol	19.59	2.31
**17**	Isopulegol	19.75	1.46
**18**	Estragole	20.60	2.70
**19**	Pulegone	21.61	58.54
**20**	Cyclohexanone, 5-methyl-2-(1-methylethylidene)	23.32	0.18
**21**	Cyclohexanone,5-methyl-2-(1-methylethylidene)-(CAS)	23.40	0.11
**22**	1,2-Cyclopropanedicarboxylic acid,3-(1-ethylethenyl)-, diethyl ester	23.46	0.22
**23**	3-Buten-2-ol,3-methyl-4-(2,6,6-trimethyl-2-cyclohexen-1-yl)-	25.44	0.19
**24**	Quercetin 7,3′,4′-Trimethoxy	54.61	0.17

**Table 5:** Chemical composition of *Ocimum basilicum* essential oil determined by GC and GC-MS.

**NO**	**Name of compounds**	**RT**	**Area **%
**1**	Eucalyptol	14.63	2.28
**2**	Trans-Sabinene Hydrate	16.19	0.59
**3**	2-Nonanone	16.87	0.50
**4**	L-Linalool	17.35	60.97
**5**	Camphor	18.65	0.81
**6**	Cyclohexanone,5-methyl-2-(1-methylethyl)-,cis- (CAS)	18.92	1.79
**7**	l-Menthone	19.20	1.16
**8**	Endo-Borneol	19.58	0.39
**9**	Terpinen-4-ol	19.77	0.15
**10**	Estragole	20.40	21.56
**11**	Pulegone	21.53	4.21
**12**	Cyclohexanone,5-methyl-2-(1-methylethylidene)	21.69	1.52
**13**	Alpha-Fenchyl acetate	22.73	1.17
**14**	Exo-2-Hydroxycineole	24.30	0.00
**15**	Trans-alpha-Bergamotene	26.74	1.57
**16**	Germacrene-D	28.07	0.66
**17**	Bicyclogermacrene	28.43	0.12
**18**	Alpha-Bulnesene	28.57	0.02
**19**	Alpha-Amorphene	28.88	0.22
**20**	Davanone	30.68	0.08
**21**	Torreyol	30.74	0.17
**22**	T-Cadinol	32.24	0.07

**Table 6:** Chemical composition of *Origanum vulgare *essential oil determined by GC and GC-MS.

**No**	**Name of compounds**	**RT**	**Area **%
**1**	Cyclopropane dodecanoic acid, 2-octyl-, methyl ester	4.26	0.01
**2**	Propane, 2,2-diethoxy	4.73	0.19
**3**	Alpha-Thujene	10.64	0.55
**4**	cis-Ocimene	10.88	2.74
**5**	Camphene	11.51	0.32
**6**	Docosahexaenoic acid,1,2,3-propanetriyl ester	11.64	0.08
**7**	Sabinene	12.46	17.17
**8**	Alpha-Myrcene	13.14	3.44
**9**	Alpha-Pinene	13.59	0.01
**10**	Geraniol formate (CAS)	13.71	0.02
**11**	Alpha-Humulene (CAS)	14.09	10.57
**12**	p-Cymene	14.42	1.35
**13**	Alpha-Phellandrene	14.57	9.18
**14**	Gamma-Terpinene	15.58	12.99
**15**	Cis-trans Sabinene hydrate	16.19	6.13
**16**	Alpha-Terpinolene	16.49	2.31
**17**	1-Terpineol	17.34	19.68
**18**	2-Cyclohexen-1-ol,1-methyl-4-(1-methylethyl)-,trans	18.01	0.43
**19**	2-Cyclohexen-1-ol,1-methyl-4-(1-methylethyl)-,cis-	18.63	0.09
**20**	5-Caranol, trans, trans-(+)-	18.92	0.12
**21**	p-Menthone	19.20	0.07
**22**	3-Cyclohexen-1-ol,4-methyl-1-(1-methylethyl)-(CAS)	19.77	5.64
**23**	Trans-Sabinene hydrate	20.36	0.18
**24**	Pulegone	21.57	0.10
**25**	Cis-sabinene hydrate acetate	21.72	6.13
**26**	Alpha-Fenchyl acetate	22.72	0.02
**27**	Caryophyllene	26.44	0.24
**28**	Gamma-Elemene	28.44	0.08
**29**	Hahnfett	52.86	0.01
**30**	Lucenin 2	53.30	0.01
**31**	Glycerine-1,3-dimyristate, 2-O-trimethylsilyl	53.55	0.05
**32**	Spirost-8-en-11-one,3-hydroxy-,(3á,5à,14á,20á,22á,25R)-	53.63	0.04
**33**	Gibberellin A19 Methyl Ester	53.72	0.02
**34**	Glycerine-1,3-dimyristate,2-O-trimethylsilyl	54.82	0.00
**35**	Quercetin 7,3′,4′-Trimethoxy	54.86	0.01
**36**	3-acetoxy-24-phenyl-4,4,14-trimethyl	56.31	0.01

## Data Availability

The data used to support the findings of this study are available from the corresponding author upon request.
